# The Potential of Metalloproteinase-9 Administration to Accelerate Mammary Involution and Boost the Immune System at Dry-Off

**DOI:** 10.3390/ani11123415

**Published:** 2021-11-30

**Authors:** Sílvia Parés, Olivia Cano-Garrido, Alex Bach, Neus Ferrer-Miralles, Antonio Villaverde, Elena Garcia-Fruitós, Anna Arís

**Affiliations:** 1 Department of Ruminant Production, IRTA (Institut de Recerca i Tecnologia Agroalimentàries), 08140 Caldes de Montbui, Spain; silviaparesriera@gmail.com (S.P.); alex.bach@icrea.cat (A.B.); 2 Institut de Biotecnologia i de Biomedicina, Universitat Autònoma de Barcelona, 08193 Cerdanyola del Vallès, Spain; olivia.cano.garrido@gmail.com (O.C.-G.); neus.ferrer@uab.cat (N.F.-M.); antonio.villaverde@uab.cat (A.V.); 3 CIBER de Bioingeniería, Biomateriales y Nanomedicina (CIBER-BBN), 08193 Cerdanyola del Vallès, Spain; 4 Departament de Genètica i de Microbiologia, Universitat Autònoma de Barcelona, 08193 Cerdanyola del Vallès, Spain; 5 ICREA (Institució Catalana de Recerca i Estudis Avançats), 08010 Barcelona, Spain

**Keywords:** dry period, mammary gland involution, MMP-9, immunity

## Abstract

**Simple Summary:**

The cow dry period is a critical period presenting a high risk of contracting intramammary infections. Active molecules to boost the innate immunity of the mammary gland and increase infection resilience could be decisive for the milking performance of dairy cows in the next lactation. Metalloproteinase-9 is a protein with a relevant role in facilitating the immune function and activating the regeneration of the mammary gland. The focus of this study was to test the role of the infusion of a recombinant version of metalloproteinase 9 at cow dry off, showing, contrary to expectations, that it is not able to enhance the innate immunity nor to improve the involution and regeneration of the mammary gland.

**Abstract:**

The dry period is decisive for the milking performance of dairy cows. The promptness of mammary gland involution at dry-off affects not only the productivity in the next lactation, but also the risk of new intra-mammary infections since it is closely related with the activity of the immune system. Matrix metalloproteinase-9 (MMP-9) is an enzyme present in the mammary gland and has an active role during involution by disrupting the extracellular matrix, mediating cell survival and the recruitment of immune cells. The objective of this study was to determine the potential of exogenous administration of a soluble and recombinant version of a truncated MMP-9 (rtMMP-9) to accelerate mammary involution and boost the immune system at dry-off, avoiding the use of antibiotics. Twelve Holstein cows were dried abruptly, and two quarters of each cow received an intra-mammary infusion of either soluble rtMMP-9 or a positive control based on immunostimulant inclusion bodies (IBs). The contralateral quarters were infused with saline solution as negative control. Samples of mammary secretion were collected during the week following dry-off to determine SCC, metalloproteinase activity, bovine serum albumin, lactoferrin, sodium, and potassium concentrations. The soluble form of rtMMP-9 increased endogenous metalloproteinase activity in the mammary gland compared with saline quarters but did not accelerate either the immune response or involution in comparison with control quarters. The results demonstrated that the strategy to increase the mammary gland immunocompetence by recombinant infusion of rtMMP-9 was unsuccessful.

## 1. Introduction

At the end of lactation and as calving approaches, pregnant dairy cows enter into a non-lactating (dry) period to optimize milk production in the subsequent lactation. During the dry period, the epithelial component of the mammary gland regresses, proliferates, and differentiates to allow optimal milk production in the subsequent lactation. Tissue regeneration of the mammary gland is necessary, and the omission of a dry period reduces milk production in the following lactation [1].

However, the strong presence of galactopoietic hormones due to a concomitant pregnancy could hamper the beginning of the involution and delay the activation of the immune system [2]. Furthermore, today’s pregnant dairy cows are dried while producing ~25 kg/d of milk with some animals producing >35 kg/d [3]. These copious amounts of milk remaining in the mammary gland exert high intra-mammary pressure and may cause discomfort [4] and milk leakage [5]. In addition, high-producing dairy cows are more susceptible to intra-mammary infections during the early stages of the dry period [6].

Involution of the mammary gland starts with a complex signaling pathway of cell factors, hormonal changes, and immune stimulation. Activation of the immune system at the beginning of the dry period recruits blood leukocytes that progressively colonize the mammary gland after dry-off and phagocytize and destroy microorganisms [7]. However phagocytic activity against pathogens is diminished at dry-off as phagocytes engulf milk fat, cell debris, and other compounds derived from milk accumulation, and their activity is not fully focused in the fight against pathogenic bacteria [7]. In fact, it is assumed that the immune system does not reach effective protective levels until 8 d after dry-off [8], and thus the health of the mammary gland is compromised during this period. To reduce the risk of mastitis, antibiotics are used routinely in the mammary gland at dry-off. However, this practice has been challenged due to concerns about the potential emergence of antibiotic resistance.

Matrix metalloproteinase-9 (MMP-9) is a tissue-remodeling enzyme that is physiologically released by mammary epithelial cells and neutrophils entering into the mammary gland during the involution process [9,10]. Thus, MMP-9 is probably one of the enzymes involved in the breaking of the extracellular matrix of the mammary gland (ECM) [11], which could trigger a signal to the detached cells to enter into apoptosis, supporting the tissue involution [12]. Moreover, the expression of MMPs, growth factors, and cytokines is closely linked, but through a mechanism that is still widely unknown in the bovine mammary gland [13,14]. It has been previously demonstrated that the proteolytic degradation of ECM is a key factor through the loss of differentiated state and induction of apoptosis and involution [15]. Thus, we hypothesized that an intramammary administration of an active fragment of the MMP-9 in a recombinant soluble form (recombinant truncated MMP-9 (rtMMP-9)) could represent an effective strategy to accelerate tissue involution at dry-off. Moreover, it could boost the infiltration of immune cells into the mammary gland and stimulate other active immune factors, reducing the need to use antibiotics at dry-off [16].

A previous study of our group tried to elucidate the effect of MMP-9 at hastening mammary gland involution by administrating rtMMP-9 at dry off [17]. However, the rtMMP-9 was not administrated in a soluble format but as inclusion bodies (IBs), which are protein nanoparticles formed spontaneously during the recombinant production process [18,19]. The IBs have some advantages compared to soluble protein, such as greater stability and slow-release of active protein. On the contrary, soluble forms present the advantage of being highly active but can sometimes be unstable and have short lives because other enzymes from the tissue can degrade them. A control of inactive rtMMP-9 IBs was also included in our last study and the conclusion was that the IB format induced an unspecific immune response, so we could not determine the MMP-9 real effect [17]. In this context, the objective of the present study is to determine the net effect of soluble rtMMP-9 compared to a saline control. A positive control at stimulating inflammation is included based on rtMMP-9 IBs.

## 2. Materials and Methods

### 2.1. Bacterial Strains, Plasmids, Recombinant Proteins and Growth Conditions

The production of rtMMP-9 was performed using *Lactococcus lactis* strain NZ9000 *clpP^−^ htrA^−^* (*clpP-htrA*; Em^R^) [20,21] (kindly provided by INRA, Jouy-en-Josas, France; patent nº EP1141337B1). The sequence of the catalytic domain of the bovine (*Bos taurus*) MMP-9 (from Phe107 to Pro449 NM_174744.2) was codon optimized for the expression in *L. lactis* and the cloning performed adding at the C-terminal a lysine plus a histidine tag to assist protein detection and purification (GeneArt, Thermo Fisher Scientific, GmbH, Regensburg, Germany). Recombinant active fragment of MMP-9 protein (39.6 kDa) was produced by expressing the encoding gene from the Cm^R^ pNZ8148 plasmid (NIZO) under the nisA promoter control [22]. This strain was cultured in shake flasks at 30 °C without shaking in M17 Broth supplemented with 0.5% glucose. Antibiotics were used at the following concentrations: chloramphenicol (5 µg/mL) and erythromycin (2.5 µg/mL). Recombinant gene expression was induced by 12.5 ng/mL nisin (Sigma-Aldrich, Barcelona, Spain) during 3 h.

### 2.2. Purification and Quantification of rtMMP-9

Pellets of *L. lactis* cells were resuspended in phosphate-buffered saline (PBS) in the presence of protease inhibitors (cOmplete™, EDTA-Free Protease Inhibitor Cocktail, Roche, Barcelona, Spain) and frozen at −80 °C. After thawing, cells were disrupted at 1500 psi (4 rounds for soluble protein and 3 for protein nanoparticles) in a French press (Thermo FA-078A). For the soluble rtMMP-9, 0.05 mg/mL lysozyme was added, and the resulting mixture was incubated at 37 °C with shaking for 2 h. The lysate was centrifuged at 15,000× *g* for 45 min. After that, soluble rtMMP-9 was recovered from the pellet as described in [23] and filtered through a 0.22 µm filter. The protein was purified by His-tag affinity chromatography using HiTrap Chelating HP 1 mL columns (GE Healthcare, Barcelona, Spain) with an ÄKTA purifier FPLC System (GE Healthcare). The purified soluble rtMMP-9 was analyzed by both SDS electrophoresis/Coomassie Brilliant Blue staining and Western blotting. Concentration was determined by Bradford’s assay (BioRad, Madrid, Spain). The isolation of rtMMP-9 IBs was conducted following previous protocols [18]. The yield of rtMMP-9 IBs was determined by both SDS electrophoresis/Coomassie Brilliant Blue staining and Western blotting, using a standard curve of known amounts of a Green Fluorescent Protein (GFP) protein. Densitometry analyses were performed with the Quantity One software (BioRad). For Western blotting, a commercial monoclonal antibody against anti-His (#A00186-100 Genescript, Piscataway, NJ, USA) and an anti-mouse secondary antibody (#170-6516 Bio Rad) were used.

### 2.3. Electron Microscopy (EM)

Purified IBs were checked by field emission scanning microscopy (FESEM) and transmission electron microscopy (TEM). In the first technique, sample microdrops were deposited 2 min on silicon wafers (Ted Pella Inc., Redding, CA, USA) and then air-dried. Nanoparticle micrographs were acquired at a nearly native state with a high-resolution in-lens secondary electron detector in a FESEM Zeiss Merlin (Zeiss, Oberkochen, Germany) operating at 2 kV. For TEM, nanoparticles samples were fixed with aldehydes and osmium, dehydrated, and embedded in Epon resin. Ultrathin sections were deposited on copper grids, and after contrast, observed with the electron microscope TEM Jeol JEM-1400 (Jeol Ltd., Tokyo, Japan).

### 2.4. Analysis of Metalloproteinase Activity

Metalloproteinase activity of rtMMP-9 (soluble MMP-9 and IBs) corresponding to −39.6 KDa bands and endogenous mammary MMP-9, corresponding to 92 KDa bands, were analyzed and compared by zymography as described elsewhere [23].

### 2.5. Intra-Mammary Infusions

This experiment was performed under the evaluation and permission of the Ethical Committee of IRTA, protocol number 9705. Twelve lactating Holstein cows (210–220 d pregnant, producing >20 kg/d of milk during the last 3 d preceding dry-off, and with milk somatic cell counts <200,000 cells/mL at dry-off) were enrolled in this study at dry-off. All cows were dried abruptly with no dietary intervention before dry-off and no changes in milking frequency to decrease the amount of milk production before stop milking. At dry-off time, two quarters of each cow received an intra-mammary infusion of either 0.75 mg of soluble rMMP-9 or 12 mg of MMP-9 nanoparticles in a total volume of 10 mL of 0.9% NaCl sterile commercial saline solution. The actual enzymatic activity of the soluble and the nanostructured MMP-9 amounts used were identical, as assessed by zymography. Treatments were randomly assigned using Excel RAND() function to front or rear quarters with soluble rtMMP-9 or IBs of rtMMP-9. The respectively contralateral quarters were infused with negative control consisting of 10 mL of 0.9% NaCl sterile commercial saline solution (Braun, Barcelona, Spain). Then, broad-spectrum antibiotics (Mamyzin secado^®^, Boehringer Ingelheim, Barcelona, Spain) were locally administered following common production practices. No teat sealant was used. A total of 50 mL of milk or mammary secretion samples were obtained at days 0 (before last milking), 1, 2, 3, 6, and 7 post-drying at 08:00 h by manual milking. A fraction of mammary secretion samples was kept refrigerated until analyzed for somatic cell counts (SCC), and the remainder was kept frozen at −20 °C until subsequent analyses.

### 2.6. Mammary Secretion Analyses

Somatic cell counts were analyzed in fresh milk (last milking before dry-off) or mammary secretion samples within 4 h after extraction using a Scepter^®^ cell counter (Merk-Millipore, Madrid, Spain) and 40 μm sensors. Briefly, 0.5 mL of mammary secretion was diluted 1:1 in PBS and cells were pelleted by centrifugation at 1000× *g* for 2 min. Cream and supernatant were discarded. Pellets were then washed 3 times in PBS and then resuspended in 0.5 mL of PBS. In some cases, samples had to be diluted in PBS prior to analysis.

Lactoferrin concentration in the last milking before dry-off and in mammary secretions was measured by ELISA, using a commercial bovine lactoferrin ELISA kit (Bethyl Laboratories Inc., Montgomery, TX, USA). The absorbance for each sample was measured at 450 nm using a Model 680 microplate reader (Bio-Rad).

The concentration of bovine serum albumin (BSA) in milk and mammary secretions was analyzed by a colorimetric assay as previously described [24], with some modifications. Briefly, 200 μL of skimmed milk or mammary secretion was mixed with 450 μL of distilled water and 450 μL of a solution containing 1 volume of 1.2 mM of bromocresol green dissolved in 5 mM NaOH, 3 volumes of 0.2 M succinic acid (pH 4.0), and 0.8% Brij 35 detergent. After mixing by inversion and centrifugation at 1900× *g* for 10 min at room temperature, 150 μL of the supernatant were added to a 96-well microplate and the optical density was read at 655 nm using a Model 680 microplate reader (Bio-Rad).

Gelatinase activity was analyzed by zymography as described above. Samples were skimmed by centrifugation at 2700× *g* for 10 min. The fat layer was discarded with a swab and supernatant was diluted between 1:20 to 1:250 in PBS and mixed 1:1 with the loading buffer.

Sodium and potassium concentrations in milk secretion were analyzed by inductively coupled plasma-Optical emission spectrometry (ICP-OES) using an ICP-OES Perkin-Elmer Optima 4300DV after dilution with Triton X-100 0.1% (*v*/*v*) instead of digested in a microwave oven as described in [25]. The method was validated before analyzing all samples by evaluating the repeatability of the results from 5 samples that were analyzed after digestion with microwaves or dilution in 0.1% (*v*/*v*) Triton X-100.

### 2.7. Statistical Analysis

Each mammary quarter was the experimental unit. All data were analyzed using a mixed-effects model that accounted for the random effects of quarter within cow, cow, and period (enrollment week), and the fixed effects of treatment, day of sampling, and their 2-way interaction. Sampling time entered the model as a repeated measure using an autoregressive covariance matrix. Metalloproteinase activity data from zymograms were previously log transformed to achieve a normal distribution. Differences were declared significant at *p* ≤ 0.05, and trends were discussed at 0.05 ≤ *p* ≤ 0.10.

## 3. Results

### 3.1. rMMP-9 Production, Activity Determination and Mammary Gland Infusion

**Figure 1:**
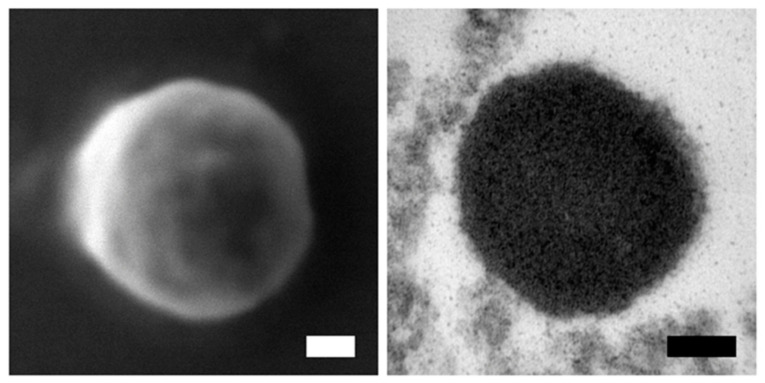
Field emission scanning electron microscopy (FESEM) micrographs of a truncated version of recombinant matrix Metalloproteinase-9 (rtMMP-9) IBs (**left panel**) and transmission electron microscopy (TEM) micrographs of rtMMP-9 IBs (**right panel**). Scale bars: 100 nm.

Truncated but active version of MMP-9 (rtMMP-9) was successfully produced in *L. lactis* either as soluble or IB. The IBs produced were used as positive control of mammary gland immunostimulation. They showed a round and compact shape with a 430 nm diameter and a smooth surface (Figure 1). When comparing the rtMMP-9 activity by zymography, the soluble form showed about 16-fold greater activity than IBs (data not shown). Equivalent metalloproteinase activity of either soluble or IB format of rtMMP-9 were infused in mammary gland quarters at dry-off.

### 3.2. Induced Metalloproteinase Activity in the Mammary Gland

**Figure 2:**
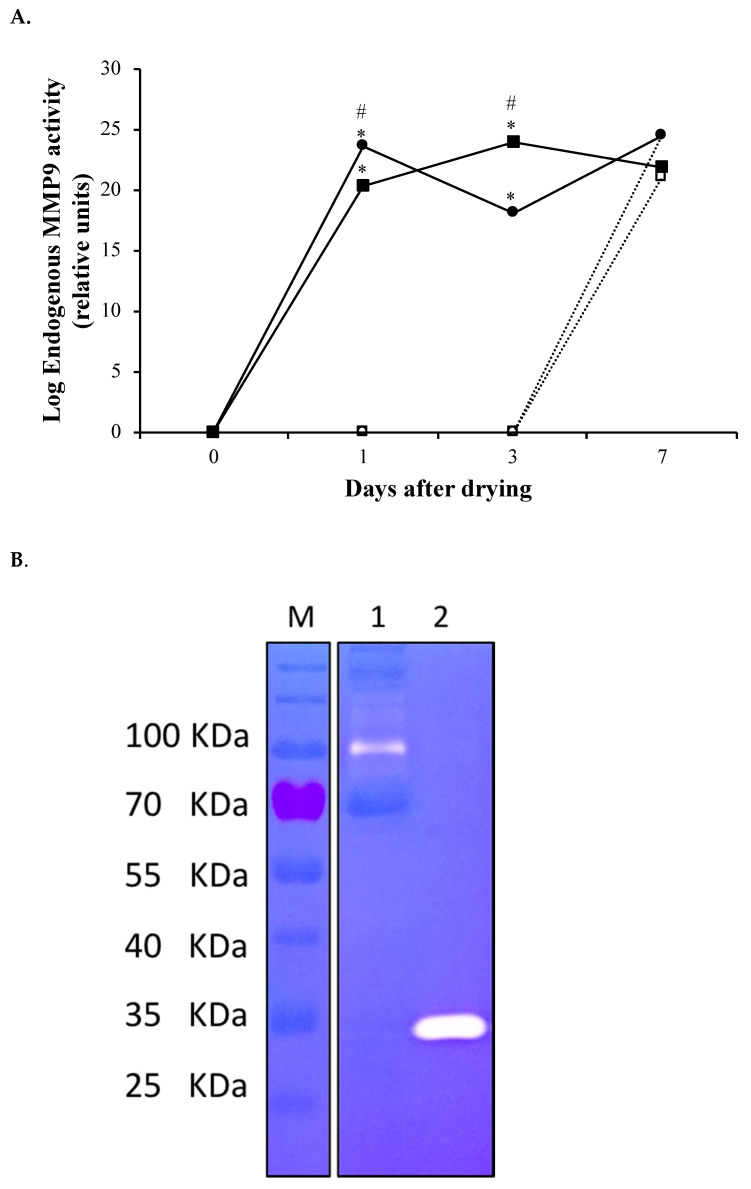
(**A**) Endogenous matrix metalloproteinase-9 (MMP-9) activity in mammary secretions at 0, 1, 3, and 7 d after dry-off of pregnant dairy-cows analyzed by zymography. Continuous lines indicate MMP-9 treatments, discontinuous lines depict controls. Filled circles correspond to soluble rtMMP-9 treatment and empty circles to its control, filled squares represent IBs treatment and empty squares to its control. Asterisks indicate significant differences (*p* < 0.05) between treatment and control whereas pound sign indicates differences between treatments. (**B**) Zymogram comparing the metalloproteinase activity (white bands) obtained in mammary secretion samples (lane 1) or loading directly the rtMMP-9 (lane 2). The Lane M corresponds to the molecular weight marker.

Metalloproteinase activity in mammary secretion was evaluated by zymography after each treatment (Figure 2). The activity of rtMMP-9 infused, either soluble or IBs, was easily differentiated from the activity corresponding to the mammary gland endogenous MMP-9 due to differences in the molecular weight between the wild type MMP-9 or rt MMP-9 (92 kDa and 39.6 kDa, respectively). Overall, endogenous metalloproteinase activity increased as days evolved since dry-off (*p* < 0.001). At days 1 and 3, the activity was greater in quarters infused with rtMMP-9 (soluble or IBs) compared with their respective saline control quarters. The activity in quarters treated with soluble rtMMP-9 was greater than in the quarters treated with IBs of rtMMP-9 at day 1. However, at day 3, the activity was greater in the IBs quarters (*p* < 0.05).

### 3.3. Mammary Immune Response and Involution Markers Monitorization after Intramammary Administration of rtMMP-9

**Figure 3:**
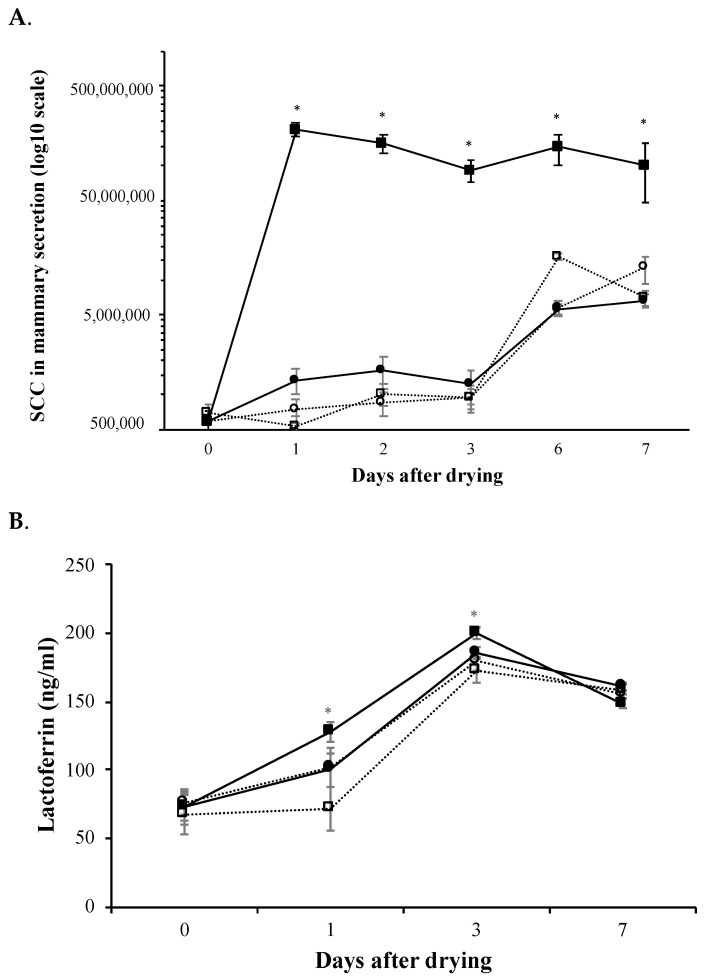
(**A**) Progression of somatic cell counts (SCC) in mammary secretion at 0, 1, 2, 3, 6 and 7 d after dry-off of pregnant dairy-cows. (**B**) Lactoferrin concentration in mammary secretion secretion at 0, 1, 3, and 7 d after dry-off of pregnant dairy-cows. In all panels continuous lines depict rtMMP-9 treatments (IBs or soluble) and discontinuous lines indicate negative controls (saline). Circles correspond to soluble rtMMP-9 treatment and to the corresponding saline contralateral quarters whereas squares represent IBs of rtMMP-9 and their negative control in contralateral quarters. Asterisks indicate significant differences (*p* < 0.05) between rtMMP-9 and negative controls.

Somatic cell counts (SCC) in mammary secretions progressively augmented (*p* < 0.0001) as time elapsed since dry-off increased (Figure 3A), but SCC in milk secretion from quarters treated with soluble rtMMP-9 or saline control did not differ (Figure 3A). However, the increase in SCC was greater at all sampling times (*p* < 0.05) in the quarters treated with positive controls (IBs of rtMMP-9) than in other treatments (Figure 3A). From day 1 to 7, SCC increased (*p* < 0.001) by 157 times in the mammary secretion from quarters infused with IBs, whereas in the quarters treated with saline solution or soluble rtMMP-9 the increase was 16 and nine times greater, respectively.

Lactoferrin concentration in mammary secretions increased (*p* < 0.0001) over time after dry-off (Figure 3B). Lactoferrin concentration in mammary secretions from quarters treated with saline control or with the soluble form of rtMMP-9 did not differ at any sampling time during the week after dry-off. However, mammary secretion from quarters treated with positive controls had a greater (*p* < 0.05) lactoferrin concentration than that from quarters with the negative control or treated with soluble rtMMP-9. There was also a positive interaction between treatment and time (*p* < 0.0001), with quarters treated with IBs of rtMMP-9 showing greater values of lactoferrin in mammary secretion at days 1 and 3 after dry-off compared with the other two treatments.

**Figure 4:**
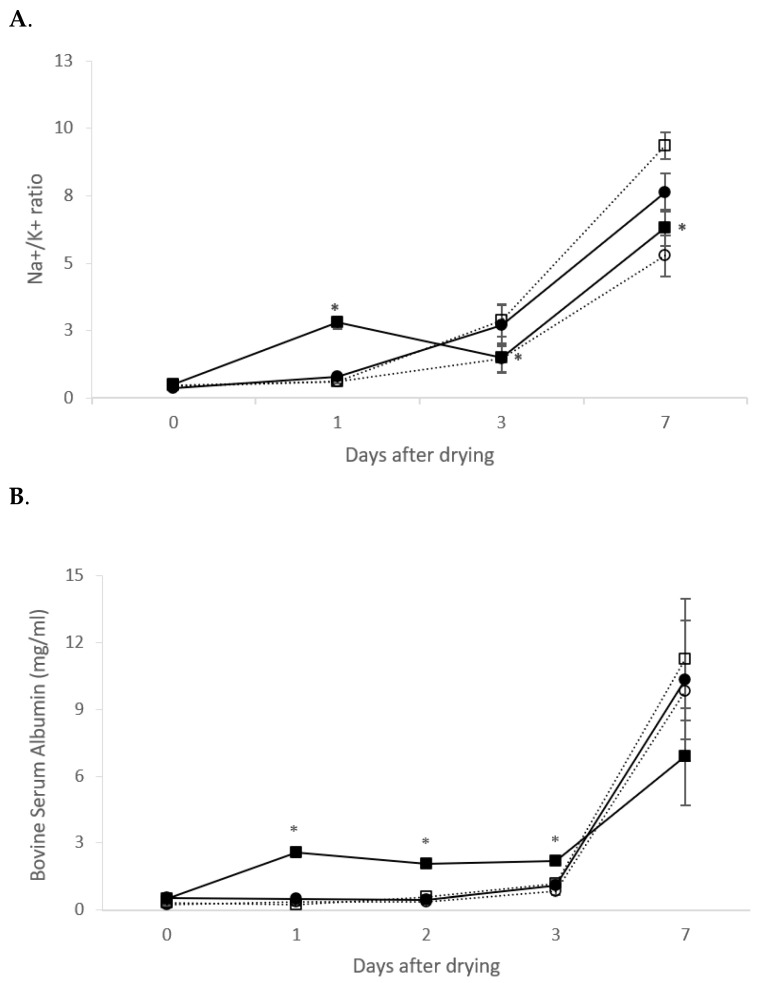
(**A**) Sodium/potassium (Na^+^/K^+^) ratio in mammary secretion at 0, 1, 3, and 7 d after dry-off of pregnant dairy-cows. (**B**) Bovine serum albumin (BSA) concentration (panel B) in mammary secretion at 0, 1, 2, 3, and 7 d after dry-off of pregnant dairy-cows. Continuous lines indicate rtMMP-9 treatments and discontinuous lines depict negative controls. Filled circles correspond to soluble rtMMP-9 treatment and empty circles to its negative control. Filled squares represent IBs of rtMMP- and empty squares to its negative control. Asterisks indicate differences (*p* < 0.05) between rtMMP-9 and controls.

Several parameters reflecting the tissular involution of the mammary gland were also evaluated (Figure 4). The sodium/potassium (Na^+^/K^+^) ratio in mammary secretion increased (*p* < 0.0001) as days since dry-off increased (Figure 4A). No differences in the Na^+^/K^+^ ratio were detected in mammary secretion between quarters treated with soluble rtMMP-9 or negative control. There was an interaction between treatment and time with mammary secretion from quarters treated with IBs of rtMMP-9 having a greater Na^+^/K^+^ ratio at day 1 compared with those treated with saline solution, but at days 3 and 7, the Na^+^/K^+^ ratio in mammary secretion from quarters treated with IBs was lower than in saline control. The presence of BSA in mammary secretion followed a similar pattern than the Na^+^/K^+^ ratio (Figure 4B), and increased (*p* < 0.0001) within days after dry-off being greater (*p* < 0.05) in the positive treatment quarters than in the other two treatments on days 1, 2, and 3 after dry-off.

## 4. Discussion

A good strategy to accelerate the cow dry period and enhance the immunocompetence of mammary gland avoiding the use of antibiotics would be to amplify the effects of some of the proteins that play important roles in the involution and remodeling processes through their exogenous administration. MMP-9 is increased during the involution of the cow mammary gland at dry-off and could potentially have a key role boosting the mammary gland involution and immune system [9]. Epithelial cells and leukocytes, like neutrophils, can express and secrete MMP-9 as a proenzyme. When secreted to the ECM, and once in its active form, MMP-9 is able to degrade cell–matrix adhesions and cell–cell integrins, increasing permeability, immune cell migration, and mammary gland involution. Although physiologically in the mammary gland MMP-9 is secreted by the local immune cells or mammary epithelia, it can also be produced by recombinant DNA technologies and infused at dry-off aiming to optimize the dry period. We have previously studied the infusion into the mammary gland of IBs as protein-based nanoparticles of active and inactive MMP-9 protein, demonstrating that IB format enhanced the involution and immune function of the cow mammary gland nonspecifically, so it was not possible to elucidate the MMP-9 effect by itself [17].

In this study, we have recombinantly produced soluble rtMMP-9 in *L. lactis* to ensure that rtMMP-9 would be free of lipopolysaccharides (LPS) and would not elicit any endotoxic response in the animal [26]. Although physiologically MMP-9 is secreted as a zymogen and then activated by proteolysis [27], we have proven that it is possible to recombinantly produce an active MMP-9 domain in soluble format [28].

The analysis of milk secretion after the infusion of rtMMP-9 in mammary gland quarters at dry-off revealed that metalloproteinase activity increased for both soluble and IB format treatments compared to negative control quarters (Figure 2). Surprisingly, we only observed in the mammary secretion a faint activity band around 35 KDa probably corresponding to the rtMMP-9. However, endogenous wtMMP-9 activity, either coming from mammary tissue or neutrophil degranulation, was clearly identified in the zymograms at 96 KDa bands. Thus, the increase in the MMP-9 endogenous activity (Figure 2) indicates that rtMMP-9, regardless of whether it is soluble or embedded in IBs, is able to activate endogenous pro-MMP-9. This is according to the fact that endogenous MMP-9 is secreted from epithelia o neutrophils as a zymogen and later activated by catalytic function or by other active metalloproteinases [29]. The activity observed at day 1 was higher for soluble rtMMP-9 treatment, while at day 3 the highest values were for rtMMP-9 IBs quarters (Figure 2), which could probably be due to a greater availability of soluble protein than the slower and more stable release of rMMP-9 in the nanoparticles [28,30,31].

Apart from the direct activity of rtMMP-9, an expanded immunostimulatory effect and/or an acceleration of mammary gland involution was assessed since it was not possible to be evaluated in previous studies only using the IBs of rtMMP-9. Herein the IBs were included as a comparative treatment, but our main focus was the effect of soluble rtMMP-9.

An equal recruitment of immune cells or SCC (Figure 3A) along with a concomitant increase in lactoferrin concentration (Figure 3B) in the soluble rtMMP-9 or negative control quarters started at day 3, achieving levels of SCC much lower (100×) than those obtained with rtMMP-9 IBs (Figure 3A). The rise in SCC in mammary secretion indicates a hastened recruitment of immune cells and the increase in lactoferrin concentration reflects a stimulation of other effectors of the innate immune system. The increased lactoferrin concentration in mammary secretions may have resulted from either an increased synthesis by mammary epithelial cells, or from a direct release by infiltrated somatic cells. In order to find an alternative to antibiotics, immunostimulation should be notably accelerated compared to the natural activation of the immune system occurring after dry-off. The reason for this is because in these first three days there is an open period when the pathogens can invade the mammary gland freely and start a decontrolled infection. In this sense, the results indicated that the activity of the soluble rtMMP-9 did not confer any positive effect speeding up the innate immunity response.

Tissue remodeling in the mammary gland was also assessed to evaluate if it was affected by the activity of exogenous rtMMP-9 administrated at dry off. The markers used to explore the tissue remodeling were BSA and Na^+^ in mammary secretion which passively diffused from blood to mammary secretion because of a tight junction’s dissociation during tissue remodeling [32]. However only an accelerated increase in both Na^+^/K^+^ ratio (Figure 4A) and BSA (Figure 4B) in mammary secretion from quarters treated with positive control (IBs) were observed, suggesting that exogenous rtMMP-9 did not affect the involution process at all [33].

The effect observed by the rtMMP-9 IBs was previously demonstrated as a result of the format of the IBs which can contain traces, such as lipids, DNA, or other proteins from the recombinant host where the protein is produced. These co-contaminants could induce an inflammatory effect in the mammary gland similar to that induced by the administration of different compounds, such as *Panax ginseng* [34], chitosan [35], and LPS [36], among others.

## 5. Conclusions

This study leads us to conclude that the hypothesis of a possible role of rtMMP-9 in accelerating the immune response and involution when exogenously administrated at dry-off is not right. The protein is functional because it induces proteolytic effects after infusion, but beyond this it is not able to affect the general resilience of the mammary gland. In this context, the possibility of using nonspecific immunostimulants, such as LPS, IBs formed by inert proteins, or some natural immunostimulants, could be an antibiotic alternative along with the further generation of antimicrobial molecules based on recombinant synthetic molecules from innate immunity.

## Data Availability

The data presented in this study are available on request from the corresponding autor.
